# Crosstalk between Akt and NF‐κB pathway mediates inhibitory effect of gas6 on monocytes‐endothelial cells interactions stimulated by *P. gingivalis*‐LPS

**DOI:** 10.1111/jcmm.15430

**Published:** 2020-05-28

**Authors:** Xuekui Wang, Yingjun Liu, Shengnan Zhang, Xiangying Ouyang, Yuguang Wang, Yong Jiang, Na An

**Affiliations:** ^1^ Department of General Dentistry II Peking University School and Hospital of Stomatology Beijing China; ^2^ Department of Periodontology Peking University School and Hospital of Stomatology Beijing China; ^3^ National Engineering Laboratory for Digital and Material Technology of Stomatology Peking University School and Hospital of Stomatology Beijing China; ^4^ National Clinical Research Center for Oral Diseases Peking University School and Hospital of Stomatology Beijing China; ^5^ Beijing Key Laboratory of Digital Stomatology Peking University School and Hospital of Stomatology Beijing China

**Keywords:** endothelial cells, gas6, GAS6‐AS2, lipopolysaccharide, *Porphyromonas gingivalis*

## Abstract

Correlation between periodontitis and atherosclerosis is well established, and the inherent mechanisms responsible for this relationship remain unclear. The biological function of growth arrest‐specific 6 (gas6) has been discovered in both atherosclerosis and inflammation. Inhibitory effects of gas6 on the expression of inflammatory factors in human umbilical vein endothelial cells (HUVECs) stimulated by *Porphyromonas gingivalis* lipopolysaccharide (*P. gingivalis*‐LPS) were reported in our previous research. Herein, the effects of gas6 on monocytes‐endothelial cells interactions in vitro and their probable mechanisms were further investigated. Gas6 protein in HUVECs was knocked down with siRNA or overexpressed with plasmids. Transwell inserts and co‐culturing system were introduced to observe chemotaxis and adhering affinity between monocytes and endothelial cells in vitro. Expression of gas6 was decreased in inflammatory periodontal tissues and HUVECs challenged with *P. gingivalis*‐LPS. The inhibitory effect of gas6 on chemotaxis and adhesion affinity between monocytes and endothelial cells was observed, and gas6 promoted Akt phosphorylation and inhibited NF‐κB phosphorylation. To our best knowledge, we are first to report that gas6 inhibit monocytes‐endothelial cells interactions in vitro induced by *P. gingivalis*‐LPS via Akt/NF‐κB pathway. Additionally, inflammation‐mediated inhibition of gas6 expression is through LncRNA GAS6‐AS2, rather than GAS6‐AS1, which is also newly reported.

## Graphical abstract

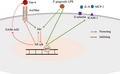

## 1 INTRODUCTION

Periodontitis is a chronic inflammatory disease caused by microbial dental plaque that eventually results in the destruction of periodontal connective tissues and alveolar bone.^1^ An association between periodontitis and atherosclerotic vascular disease has previously been validated.^2^ Among the possible mechanisms involved in this association, systemic and/or local inflammation mechanisms have been studied most recently.^3^, ^4^ In the aforementioned study of periodontitis, the bacterial plaque destroyed the epithelium of the periodontal pocket, allowing the entry of harmful elements (such as endotoxins, exotoxins and bacteria) into the bloodstream, causing a low‐grade, systemic inflammatory condition. In addition to the direct invasion of the vessel wall, the presence of oral pathogens such as *P. gingivalis* (one of the key periodontal pathogens) was observed.^5^ In our previous study, DNA from *P. gingivalis* was also found in human atheromatous plaques.^6^ A separate study showed that pathogenic substances leaking into blood vessels triggered an inflammatory response that lead to endothelial dysfunction,^7^ an important contributor to the initiation and progression of atherosclerotic lesions.^8^ Such inflammatory responses are often due to the presence of lipopolysaccharides.

LPS—also known as lipoglycans and endotoxins—are found in the outer membranes of Gram‐negative bacteria and are one of their key virulence factors. In previous studies, elevated LPS level circulating in the blood of periodontitis patients have been related to an increased risk of atherosclerosis.^9^ Endothelial dysfunction has also been observed in blood vessels stimulated by LPS from periodontal pathogens.^10^, ^11^ In our previous report, *P. gingivalis*‐LPS stimulation not only negatively affected the viability, proliferation and migration of HUVECs, but also positively promoted the secretion of adhesion molecules (ICAM‐1, E‐selectin) and chemokines (MCP‐1, IL‐8) in vitro*,* ^12^ which promoted local leucocyte infiltration and prompted the occurrence of atherosclerotic lesions.

Growth arrest‐specific 6 (gas6)—a 75KD‐secreted protein first found in serum‐starved NIH 3T3 cells by Manfioletti in 1993—belongs to the vitamin K‐dependent protein family. The gas6 protein shares a 43% amino acid identity with protein S—a negative coregulatory molecule involved in blood coagulation pathways. Gas6 contains an N‐terminal carboxy glutamic acid (Gla) domain—a region rich in glutamic acid residues that is γ‐carboxylated in a vitamin K‐dependent reaction. In addition, gas6 contains four epidermal growth factor‐like domains and two laminin globular‐like domains that contain the interaction sites for its TAM (Tyro3, Axl and Mer) receptor tyrosine kinases.^13^ Gas6 is ubiquitously expressed in many cells including endothelial cells. The gas6/TAM system participates in several pathophysiological processes including thrombosis, the phagocytosis of apoptotic cells, inflammation inhibition and vascular calcification.^14^ TAM‐dependent pathways act as a negative feedback mechanism that suppresses inflammation.^15^ Gas6 is a key homeostatic, immunological regulator of host‐commensal interactions in the oral mucosa. The absence of gas6 has been shown to increase the anaerobic bacterial load and, consequently, the level of gingival inflammation in vivo.^16^ In the context of atherosclerosis, Axl and Tyro3 are down‐regulated in advanced human carotid plaques,^17^ while Mer mutations promoted the necrosis of atherosclerotic plaques in ApoE^‐/‐^ mice.^18^ Additionally, gas6 has been independently associated with reduced plaque height and total plaque area.^19^ Protective effects of Gas6 on endothelial tight junction and permeability were also recently demonstrated in vivo.^20^ Together, these data illustrate the critical role of gas6 in inflammation and atherosclerosis, and show that gas6 is likely the base molecule of the mechanisms underlying the association between periodontitis and atherosclerosis.

The earliest pathological changes of atherosclerosis involve the activation of endothelial cells, which recruit monocytes and then tether them to the intima. We observed that gas6 exerted an inhibitory effect on the mRNA expression of adhesion molecules and chemokines in HUVECs stimulated with 1μg/mL *P. gingivalis*‐LPS.^21^ However, the influence and mechanisms of gas6 on the recruiting and adhering functions of the HUVECs remained unclear. Therefore, the aims of this study were to: (a) observe the in vitro effect of gas6 on chemotaxis and adhesion of monocytes to HUVECs stimulated by *P. gingivalis*‐LPS and (b) explore the possible mechanisms of gas6 involved in this process.

## 2 MATERIALS AND METHODS

### 2.1 Cell culture

HUVECs (ScienCell) were cultured in endothelial culture medium (ScienCell) containing 10% foetal bovine serum (FBS), 1% endothelial cell growth supplements, 100 IU/mL penicillin and 100 μg/mL of streptomycin. Human monocytic cell line THP‐1 (ATCC) cells were cultured in RPMI 1640 basic medium (Gibco) supplemented with 10% foetal bovine serum, 100 IU/mL penicillin and 100 μg/mL of streptomycin. Cultures were maintained at 37℃ in an incubator containing a humidified mixture of 95% air and 5% CO_2_. HUVECs subcultured at passages 3‐5 were used in the following experiments. Ultra‐pure *P. gingivalis*‐LPS was purchased from InvivoGen and dissolved in endotoxin‐free water at a concentration of 1 mg/mL; the resulting solution was stored at −20°C. LPS preparations were free from lipoproteins as reported by other study.^22^

### 2.2 Cell transfection

HUVEC cultures reaching 50%–70% confluence were transfected with gas6 siRNA (si‐Gas6) with a scrambled siRNA (si‐CTR) as a negative control to knock‐down gas6 expression—or with pcDNA3.1(+) plasmids to overexpress gas6. To knock‐down the expression level of GAS6‐AS2, plasmids containing Gas6‐AS2 short hairpin RNA (sh‐Gas6‐AS2) were used. Delivery of siRNAs, shRNAs or plasmids in this study was performed with a Lipofectamine 3000 Transfection Kit (Invitrogen). Transfection efficiency was established by determining the expression level of either gas6 or GAS6‐AS2 by real‐time qPCR and Western blot assays.

### 2.3 Real‐time PCR

Total RNA was isolated using TRizol reagent (Thermo Fisher Scientific) and reverse transcribed to cDNA according to the manufacturer's instructions. This mix (containing total cDNA, forward and reverse primer, Milli‐Q water and SyberGreen reagent (Roche)) was subjected to thermal cycling performed in a 7500 Fast Time Real‐Time PCR system (Applied Biosystems). PCR results were analysed using the 2^‐ΔΔCT^ method and presented as the relevant expression level, as normalized to the level of housekeeping gene GAPDH. All samples were amplified in duplicate, and all experiments were repeated three times. The primers used in this study were summarized on Chart 1 in Supporting Information.

### 2.4 Western blot analysis

Total cellular or tissue protein was homogenized in highly efficient RIPA buffer (Solarbio) supplemented with a 1% complete protease inhibitor cocktail (Sigma‐Aldrich) and, when necessary, phosphatase inhibitors. After sonication and centrifugation of the cell lysates, proteins in the supernatant were determined via BCA assay (Solarbio) and resolved on an 8% SDS‐PAGE gel at 20‐30 µg per lane as appropriate. These gels were electro‐transferred onto a PVDF membrane. Transfer was followed by antibody blocking of the membrane with 5% skim milk for 1 hour, incubation of the first antibody overnight at 4°C and subsequent HRP‐conjugated second antibody incubation for 1 hour at room temperature. The primal antibodies used in this study were as follows: Phospho‐Akt (Ser473) Rabbit mAb, NF‐κB p65(D14E12) Rabbit mAb, GAS6 (D3A3G) Rabbit mAb, Phospho‐NF‐κB p65 (Ser536) Rabbit mAb, CD54/ICAM‐1 Rabbit Antibody, GAPDH (D16H11) Rabbit mAb (CST), Anti‐pan‐AKT Rabbit Antibody (Abcam), Rabbit Anti‐AXL Polyclonal Antibody, Rabbit Anti‐E‐selectin Polyclonal Antibody (Bioss), TYRO3 Polyclonal Antibody Rabbit (Abclonal) and the Rabbit MERTK Antibody (CUSABIO). The target proteins’ blot signal was revealed by chemiluminescence and quantified by densitometry using the ImageJ software 1.46r. Results were expressed as a relative expression normalized to GAPDH level.

### 2.5 Monocyte chemotaxis assay

HUVECs were seeded on basal side of 8.0‐µm cell culture transwells (Corning) at a density of 3 × 10^4^ cell/well. After these cultures reached full confluence, the following concentrations of *P. gingivalis*‐LPS were used to stimulate the plated HUVEC for 24 hours: 0 μg/mL, 0.1 μg/mL, 1 µg/mL and 10 µg/mL. When the effects of gas6 were observed, 1 μg/mL *P. gingivalis*‐LPS were used to stimulate conditioned HUVECs for 24 hours. Then, THP‐1 cells, pre‐labelled with 20 µM Calcein AM for 30 minutes, were seeded onto apical side of the chamber (conc: 1 × 10^5^ cell/well). Monocytes were observed transmigrating to the basal chamber after 3 hours using a Zeiss inverted microscope. Three of these images were randomly selected for analysis.

### 2.6 Monocyte adhesion assay

HUVECs (1 × 10^5^ cells/well) were seeded onto 12‐well cell culture plates and allowed to form a cell monolayer. The cells were then stimulated by varying concentrations (0 µg/mL, 0.1 µg/mL, 1 µg/mL and 10 µg/mL) of *P. gingivalis*‐LPS for 24 hours. In culture wells where the gas6 siRNA and overexpression plasmids were used, 1 μg/mL *P. gingivalis*‐LPS was used to stimulate conditioned HUVECs for 24 hours. The culturing medium was replaced with fresh endothelial medium to eliminate the influence of LPS on monocytes added later.

THP‐1 cells (5 × 10^5^ cell/well) pre‐labelled with 20 μM Calcein AM for 30 minutes were co‐cultured with HUVECs for 4 hours. PBS was used to gently wash non‐adherent THP‐1 cells thrice; THP‐1 cells that adhered to the surface of HUVECs were photographed using a Zeiss inverted microscope. Three of these images were randomly selected for analysis.

### 2.7 Patients and tissue samples

Six healthy gingival specimens (H1‐H6) containing both epithelium and connective tissue were obtained during crown lengthening surgery. Four inflammatory periodontal tissues (I1‐I4) were obtained during periodontal debridement and flap surgery. The inclusion criteria were (a) diagnosed with periodontitis and indicated for periodontal flap surgery (bleeding on probing and probing depth ≥ 5 mm after initial therapy), (b) indicated for crown lengthening surgery with a probing depth ≤ 3 mm and BOP (bleeding on probing) was negative at surgical site. Exclusion criteria included systemic diseases—such as diabetes mellitus or any metabolic syndrome affecting periodontal tissues, antimicrobial or medicinal treatment in the previous 6 months, history of smoking, and (in women) pregnancy or lactation. This study was conducted in accordance with the Declaration of Helsinki and was approved by the Ethics Committee of Peking University School and Hospital of Stomatology (PKUSSIRB‐201948107). All participants gave their written informed consent. Tissues were rinsed with PBS to remove blood contamination and cryopreserved at −80°C immediately until further use. Tissues were cut into small pieces with sterile scissors and lysed using a Schwing mühle TissueLyser 2 (Qiagen). A Western blotting assay was used to analyse the total protein extracted from these tissues. Immunodetection and qualification of gas6 was performed with antibodies against GAPDH and gas6 in 1:1000 dilution.

### 2.8 Statistical analysis

All experiments were performed in triplicate. Results were expressed as means ± SE. An unpaired two tailed t test was performed to analyse data from two groups, and one‐way analysis of variance (ANOVA) was performed to analyse data involving more than two groups. Significance analysis of data from healthy and inflammatory periodontal tissues was also performed with a t test. Values of *P* ≤ .05 were considered statistically significant. All data analysis was performed with SPSS software 21.0 (SPSS Inc). Corresponding symbols in figures are * for *P* < .05, ** for *P* < .01 and *** for *P* < .001.

## 3 RESULTS

### 3.1 Chemotaxis and adhesion of monocytes to HUVECs was promoted by *P. gingivalis*‐LPS stimulation

**Figure 1:**
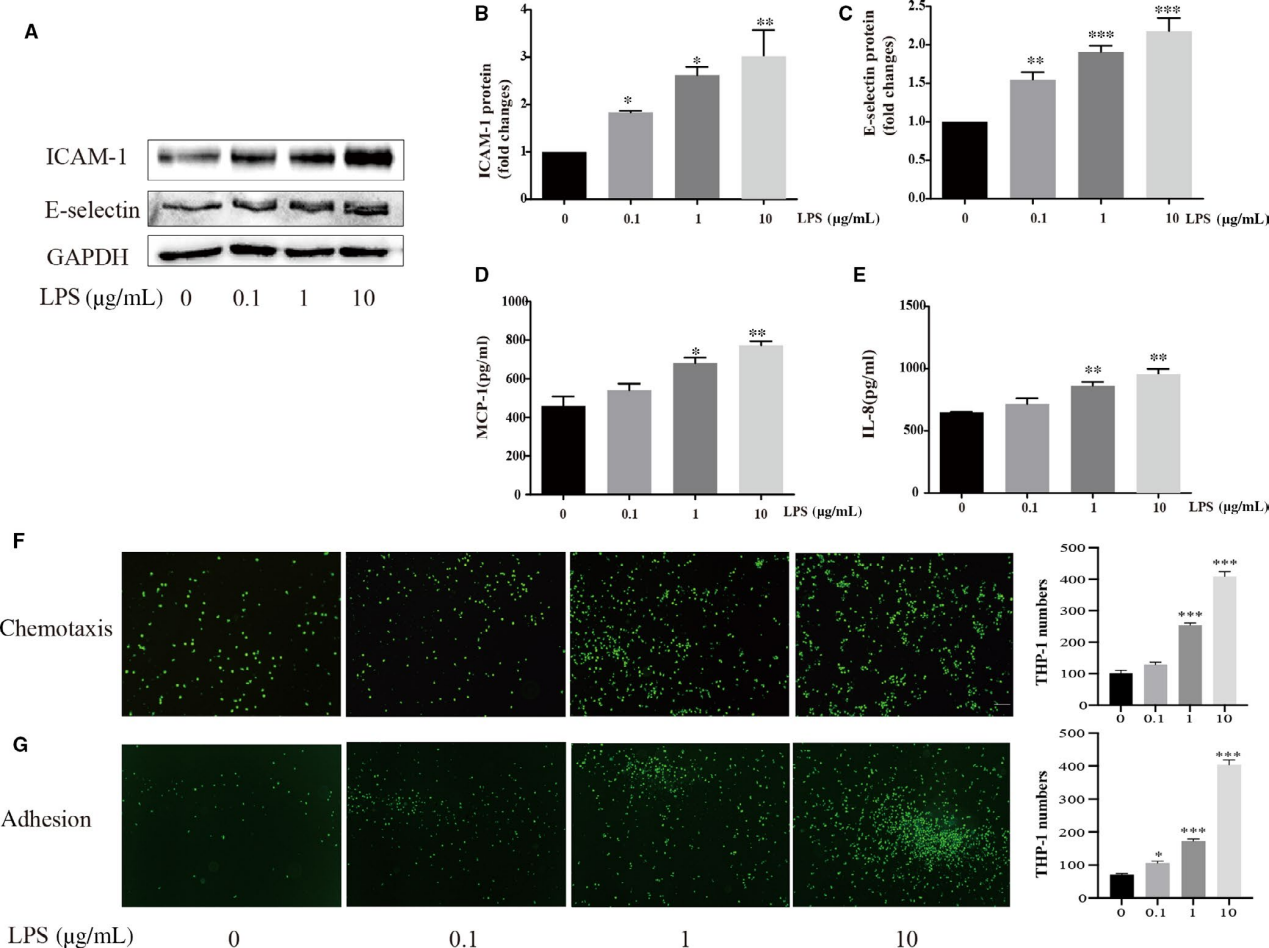
Effect of *P. gingivalis*‐LPS on chemotaxis and adhesion of monocyte to HUVECs. (A‐C) expression of adhesion molecules, ICAM‐1 and E‐selectin, in HUVECs challenged with different concentration of *P. gingivalis*‐LPS for 24 hours. (D‐E) expression of chemokines, IL‐8 and MCP‐1, in HUVECs challenged with different concentration of *P. gingivalis*‐LPS for 24 hours were measure with ELISA method. Unpaired Student's t test was performed (B‐E). *P < .05, **P < .01 and ***P < .001 vs 0 μg/mL group. (F) representative images (3 independent experiments) showing monocytes recruited by HUVECs, HUVECs in lower chamber of transwell culture system were stimulated with different concentration of *P. gingivalis*‐LPS for 24 hours, images were captured 3 hours after THP‐1 cells were added into the upper chambers. Scale bars, 100 μm. (G) representative images (3 independent experiments) showing monocytes adhering to the surfaces of HUVECs. Endothelial cells were cultured in 6‐well plates and stimulated with different concentration of *P. gingivalis*‐LPS for 24 hours, THP‐1 cells were co‐cultured with endothelial cells for 3 hours, images were captured after non‐adherent monocytes were rinsed out gently with PBS for 3 times. Scale bars, 100 μm

The effect of *P. gingivalis*‐LPS infection on the expression of chemokines and adhesion molecules in HUVECs is shown in Figure 1. The protein levels of ICAM‐1 and E‐selectin were significantly elevated compared to the negative control (*P* < .05) in HUVECs following LPS stimulation—in all three experimental concentrations. In addition, no difference in the protein level of MCP‐1 or IL‐8 was observed between the 0.1 μg/mL *P. gingivalis*‐LPS experimental group and the control group (*P* > .05). Figure 1F shows images of THP‐1 cells recruited by HUVECs in the transwell system after stimulation by the various experimental concentrations of *P. gingivalis*‐LPS. Monocytic chemotaxis towards HUVECs was observed to be facilitated by *P. gingivalis*‐LPS in a dose‐dependent manner. Similar effects of *P. gingivalis*‐LPS were noted on the number of THP‐1 cells that adhered to the surfaces of HUVECs, which is also demonstrated in Figure 1G.

### 3.2 Gas6 inhibited *P. gingivalis*‐LPS induced chemotaxis of monocytes towards HUVECs *in vitro*

**Figure 2:**
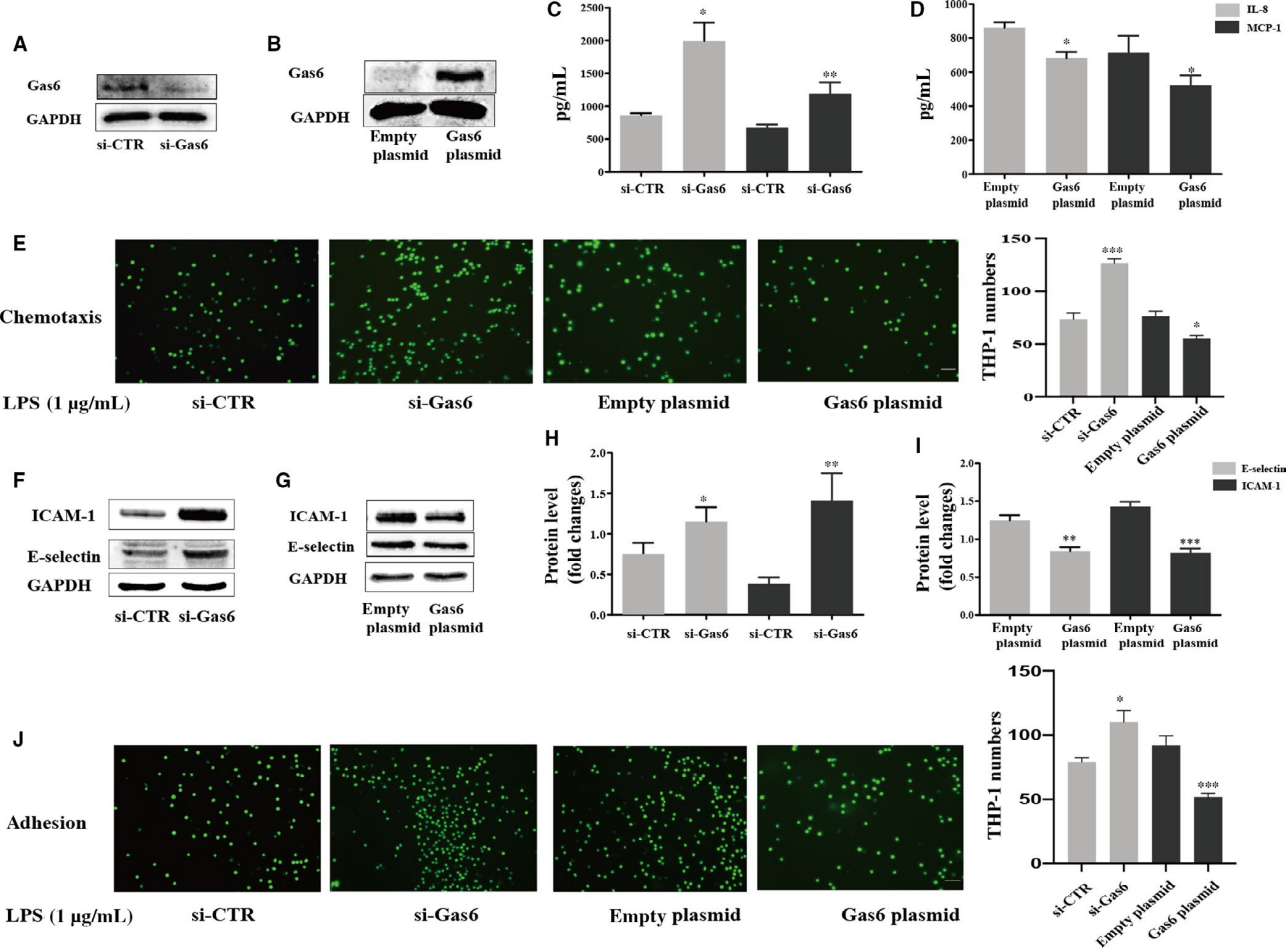
Effect of gas6 in HUVECs on chemotaxis and adhesion between monocytes and endothelial cells stimulated by *P. gingivalis*‐LPS. (A‐B) Western blotting for checking efficiency of gas6 transfection in HUVECs. (C‐D) expression of chemokines MCP‐1 and IL‐8 in HUVECS transfected with gas6 siRNA or plasmids, followed with 1 μg/mL *P. gingivalis*‐LPS infection for 24 hours. Expression level were detected by ELISA method. *P < .05, **P < .01 and ***P < .001 vs indicated control groups. (E) representative images (3 independent experiments) showing monocytes recruited by endothelial cells. Gas6 siRNA or plasmid were transfected into HUVECs in the lower chamber of transwell inserts. HUVECs were challenged with 1 μg/mL *P. gingivalis*‐LPS for 24 hours, images were captured 3 hours after Calcein AM pre‐labelled THP‐1 cells were added into the upper chamber. Scale bars, 200 μm. (F‐I) Western blotting for detection of adhesion molecules ICAM‐1 and E‐selectin in HUVECS transfected with gas6 siRNA or plasmids, followed with 1 μg/mL *P. gingivalis*‐LPS infection for 24 hours. *P < .05 vs indicated control groups. (J) representative images (3 independent experiments) showing monocytes adhering to the surfaces of HUVECs. HUVECs were transfected with gas6 siRNA or plasmid, followed with 1 μg/mL *P. gingivalis*‐LPS stimulation for 24 hours. THP‐1 cells were co‐cultured with HUVECs for 3 hours, images were captured after non‐adherent monocytes were removed gently with PBS for 3 times. Scale bar, 200 μm. Unpaired Student's t test was performed (C‐D, H‐I)

As shown in Figure 2A‐B, gas6 expression in HUVECs was efficiently knocked down or overexpressed. The effect of gas6 on chemokine expression in *P. gingivalis*‐LPS stimulated HUVECs was shown in Figure 2C‐D. HUVECs that underwent gas6 knock‐down also displayed increased levels of MCP‐1 and IL‐8 (compared to HUVECs that underwent *P. gingivalis*‐LPS stimulation alone (*P* < .05), while the levels of these chemokines were conversely decreased (*P* < .05) in HUVECs that experienced gas6 overexpression. The effect of gas6 on chemotaxis within HUVECs (in vitro) was shown in Figure 2E. After gas6 was knocked down and these cells underwent *P. gingivalis‐*LPS stimulation, the number of THP‐1 monocytes that migrated towards endothelial cells was considerably increased. Conversely, an inhibitory effect on chemotaxis was observed after gas6 was overexpressed in HUVECs.

### 3.3 Gas6 inhibited monocytes‐endothelial cells adhesion stimulated by *P. gingivalis*‐LPS in *vitro*

ICAM‐1 and E‐selectin expression exhibited an increase when gas6 was knocked down in HUVECs; the opposite effect was observed in the gas6 overexpression group (Figure 2F‐I). Similarly, gas6 knock‐down in HUVECs—combined with *P. gingivalis*‐LPS stimulation—further promoted the adherence of monocytes to the HUVECs’ surface, whereas the adhering ability of HUVECs was reduced in response to *P. gingivalis*‐LPS when gas6 was overexpressed (Figure 2J). In summary, Gas6 in HUVECs inhibited monocytes‐endothelial interactions promoted by *P. gingivalis*‐LPS infection.

### 3.4 Both Axl and Mer receptors participated into the inhibitory effect of gas6

**Figure 3:**
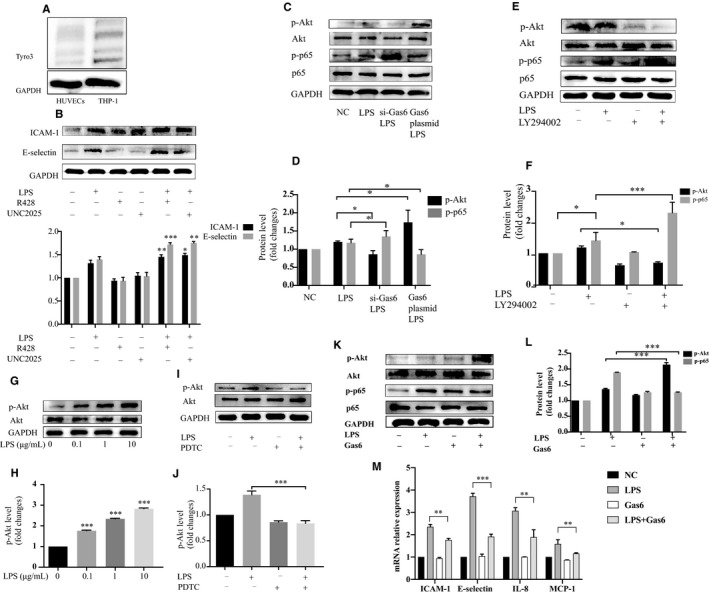
Akt/NF‐κB pathway mediated the gas6 inhibitory effect. (A) Western blotting for detection of Tyro3 receptor in HUVECs, THP‐1 cells were set as positive control. (B)Western blotting for detecting change of ICAM‐1 and E‐selectin protein level in HUVECs pre‐incubated with selective small molecular inhibitors of Axl and Mer receptor, R428 (10 μg/mL) and UNC2025 (10 μM), respectively, followed by challenged with 1 μg/mL *P. gingivalis*‐LPS. (C‐D) change of phosphorylated p65 or Akt level when gas6 in HUVECs was knock‐down or overexpressed, followed with 1 μg/mL *P. gingivalis*‐LPS stimulation for 3 hours. (E‐F) change of phosphorylated p65 or Akt level in HUVECs pre‐treated with 30 μM LY294002 for 1 hour and stimulated by 1 μg/mL *P. gingivalis*‐LPS for 3 hours. (G‐H) expression of phosphorylated Akt in HUVECs challenged with 0 μg/mL, 0.1 μg/mL, 1 μg/mL and 10 μg/mL for 3 hours. (I‐J) change of phosphorylated Akt level in HUVECs pre‐treated with 100 μM PDTC for 1 hour and challenged with 1 μg/mL *P. gingivalis*‐LPS. (K‐L) expression of phosphorylated p65 and Akt in HUVECs pre‐treated with 400 ng/mL recombinant human gas6 protein for 1 hour and stimulated with 1 μg/mL *P. gingivalis*‐LPS for 3 hours. (M) mRNA level of ICAM‐1, E‐selectin, MCP‐1 and IL‐8 in HUVECs pre‐treated with 400ng/mL recombinant human gas6 protein for 1 hour, followed by stimulation with 1 μg/mL *P. gingivalis*‐LPS for 24 hours. One‐way ANOVA analysis was performed. ( *P < .05, **P < .01 and ***P < .001)

As shown in Figure 3A, Tyro3 expression within the HUVECs was not detected by Western blotting assays, and further analysis on Tyro3 receptor was therefore precluded. Axl and Mer receptors were blocked with selective small molecular inhibitors, R428 (10 μg/mL) and UNC2025 (10 nM), respectively. ICAM‐1 and E‐selectin expression in HUVECs were significantly elevated compared to *P. gingivalis*‐LPS stimulation alone (Figure 3B, *P* < .05).

### 3.5 Akt/NF‐κB pathway mediated gas6 inhibitory effect

Gas6 knock‐down, followed by stimulation with *P. gingivalis*‐LPS, significantly inhibited the expression of phosphorylated Akt and promoted phosphorylated p65 expression (*P* < .05) within HUVECs; expression changes of phosphorylated Akt and phosphorylated p65 were reversed in HUVECs over‐expressing gas6 (p＜0.05, Figure 3C‐D). Phosphorylated Akt levels were efficiently inhibited in HUVECs pre‐incubated with a broad‐spectrum inhibitor of PI3K (30 µM LY294002). This inhibition of Akt activation boosted the level of phosphorylated p65 (p＜0.001, Figure 3E‐F). On the other hand, phosphorylated Akt levels in HUVECs stimulated by *P. gingivalis*‐LPS was elevated in a dose‐dependent manner (*P* < .001) (Figure 3G‐H), and inhibition of phosphorylated Akt was also observed after the phosphorylated p65 expression was inhibited (*P* < .001, Figure 3I‐J). Additionally, elevated expression of phosphorylated Akt and decreased expression of phosphorylated p65 were detected in HUVECs when exogenous recombinant human gas6 protein was introduced (*P* < .001, Figure 3K‐M), and also, levels of chemokines and adhesion molecules were decreased accordingly, which mirrored the effect when gas6 was overexpressed. All data combined, Akt/NF‐κB pathway mediated gas6 inhibitory effect on monocytes‐endothelial cells interactions induced by *P. gingivalis*‐LPS.

### 3.6 Expression of gas6 was decreased in *P. gingivalis*‐LPS stimulated HUVECs

**Figure 4:**
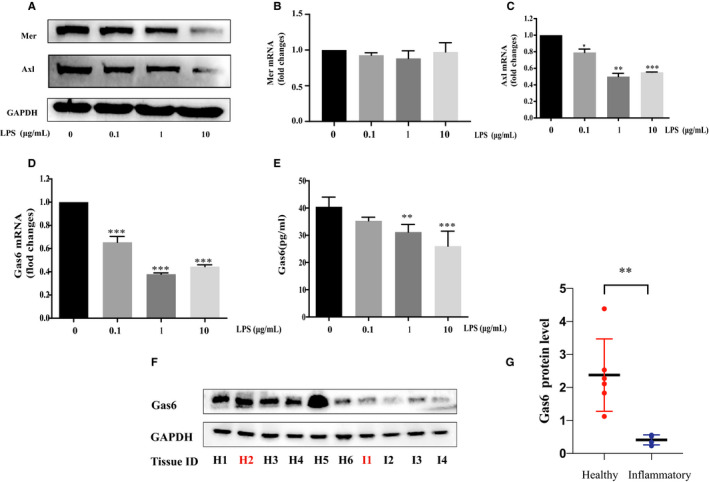
Effects of *P. gingivalis*‐LPS on expression of gas6 and its receptors in HUVECs. (A‐E) mRNA and protein level of gas6, Axl and Mer in HUVECs challenged with 0 μg/mL, 0.1 μg/mL, 1 μg/mL and 10 μg/mL *P. gingivalis*‐LPS for 24 hours. (F‐G) Western blot assay for detecting gas6 protein levels in healthy (H1‐H6) and inflamed (I1‐I4) periodontal tissues. Note that H2 and I1 tissue are from the same patient at the same time. Unpaired Student's t test was performed (B‐G). (*P < .05, **P < .01 and ***P < .001)

The expression of gas6 and receptors, Axl and Mer, in HUVECs when challenged with *P. gingivalis*‐LPS at different concentrations were investigated. Both mRNA and protein results indicated that *P. gingivalis*‐LPS down‐regulated the expression of both gas6 and its receptor Axl (Figure 4A,C‐E, *P* < .05). However, no difference in Mer mRNA expression level was found between four groups (Figure 4B, *P* > .05). Analysis of gas6 protein expression in periodontal tissues from healthy and periodontitis‐compromised patients demonstrated a similar result, as gas6 protein levels were obviously reduced in the compromised groups (*P* < .01, Figure 4F‐G). It should be noted that the H2 and I1 tissue samples were from the same patient (the H2 tissue was collected during crown lengthening surgery at right mandibular first and second molars, and the I1 tissue was collected from distal wedge flap surgery at right mandible). Probing depth at the distal site of the second molars was nearly 7 millimetres and bleeding was also present on probing, indicating an inflammatory condition. Altogether, gas6 level was decreased in HUVECs and periodontal tissue under inflammatory conditions.

### 3.7 LncRNA GAS6‐AS2 mediated the inhibitory effect of *P. gingivalis*‐LPS on gas6 expression

**Figure 5:**
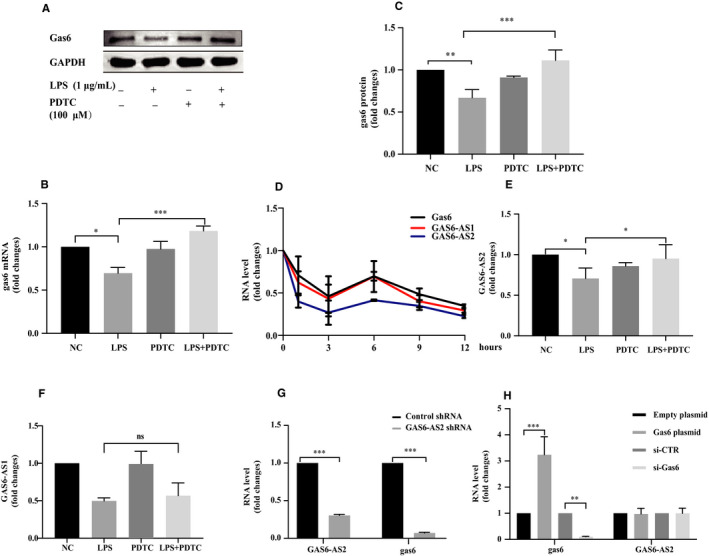
The inhibitory effect of *P. gingivalis*‐LPS on gas6 expression in HUVECs is mediated by LncRNA GAS6‐AS2 down‐regulation. (A‐C) inhibited mRNA and protein expression of gas6 caused by 1μg/mL *P. gingivalis*‐LPS was reduced by pre‐treating HUVECs with 100 μM PDTC for 1 hour. (D) expression of gas6 mRNA, GAS6‐AS1 and GAS6‐AS2 in HUVECs after stimulated with 1 μg/mL *P. gingivalis*‐LPS for 1, 3, 6, 9 and 12 hours. (E‐F) Expression of GAS6‐AS1 and GAS6‐AS2 in HUVECs after pre‐treatment with PDTC for 1 hour and stimulation with 1 μg/mL *P. gingivalis*‐LPS for 24 hours. (G) expression of gas6 in HUVECs after GAS6‐AS2 was knocked down with shRNA. (H) expression of GAS6‐AS2 in HUVECs after gas6 was knocked down or overexpressed. One‐way ANOVA analysis was performed (B‐F). Unpaired Student's t test was performed (G‐H). (ns: not significant, *P < .05, **P < .01 and ***P < .001)

Effect of *P. gingivalis*‐LPS mediated inhibition of gas6 expression in HUVECs was abolished when HUVECs was pre‐treated with Pyrrolidinedithiocarbamate ammonium (PDTC), the selective inhibitor of NF‐κB (Figure 5A‐C). As depicted in Figure 5D, the expression level changes of two antisense RNAs, GAS6‐AS1 and GAS6‐AS2, were consistent with gas6 expression in 1μg/mL *P. gingivalis*‐LPS challenged HUVECs at different time. In the presence of PDTC, *P. gingivalis*‐LPS ‐mediated down‐regulation of GAS6‐AS2 expression was reversed, a finding consistent with gas6 expression (Figure 5E), while GAS6‐AS1 expression remained unaffected (Figure 5F, *P* > .05). Gas6 expression was likewise inhibited when GAS6‐AS2 was knocked down using GAS6‐AS2 shRNAs (Figure 5G); no difference in GAS6‐AS2 expression level was observed after gas6 was knocked down or overexpressed (Figure 5H, *P* > .05), from which we can conclude that GAS6‐AS2 was an upstream regulatory factor for gas6 expression.

## 4 DISCUSSION

In this study, we found that gas6 protein within HUVECs inhibited the chemotaxis and adhesion of monocytes to endothelial cells stimulated by *P. gingivalis*‐LPS. LncRNA GAS6‐AS2, rather than GAS6‐AS1, mediated the inhibitory effect of NF‐κB on gas6 expression as was first reported.

The initial pathological process of atherosclerosis is characterized by circulating monocytes being recruited to dysfunctional endothelial cells, adhering to their surfaces and transmigrating the endothelium into the intima. Classic chemokines (MCP‐1, IL‐8) and adhesion molecules (ICAM‐1, E‐selectin) participated in aforementioned pathological processes were observed in the present study. LPS from *P. gingivalis* significantly promoted (in a dose‐dependent manner) the expression of chemokines and adhesion molecules. To directly investigate the effects of *P. gingivalis*‐LPS on the interactions between monocytes and endothelial cells, migration assays within transwell inserts and a co‐culture system were introduced. The chemotaxis and adhesion of monocytes to HUVECs were clearly enhanced when stimulated with *P. gingivalis*‐LPS, which is congruent with other studies.^23^, ^24^ While THP‐1 cells used in our experiments as monocytes was not so good as reflecting pathophysiological relevance of the results, THP‐1 is a well‐known and frequently used monocyte cell line for its homogeneous genetic background, shorter doubling time, and most importantly, relatively similar response patterns compared with human peripheral blood monocytes.^25^

The TAM‐dependent pathways lie at the intersection of the innate and adaptive immune systems, where they provide inhibitory feedback that is required to dampen inflammation.^14^ Inhibition of Toll‐like receptor‐driven inflammation exerted by gas6/TAM system was first demonstrated by Rothlin et al^15^ LPS used in our study is a ligand for Toll‐like receptor, and to elucidate the effects of gas6, siRNA and plasmids were introduced. Knock‐down of gas6 (followed by 1 μg/mL *P. gingivalis*‐LPS infection) resulted in further increased chemokine and adhesion molecule levels within the HUVECs, whereas overexpression of gas6 efficiently inhibited the cytokines expression. Accordingly, monocyte chemotaxis and adhesion were mitigated by gas6. Previous studies^26^, ^27^, ^28^, ^29^ showed that several aspects of gas6/TAM systems are involved in orchestrating inflammation, including the attenuation of inflammatory lung injury and sepsis‐induced tight junction injury. Congruent with our findings, gas6 was also reported to inhibit the adhesion of polymorphonuclear cells to endothelial cells in a dose‐dependent manner,^30^ and not only inhibit VCAM‐1 expression in human microvascular endothelial cells induced by high glucose,^31^ but inhibit NF‐κB activation in mouse aortic endothelial cells induced by *E. coli* LPS.^32^

However, our results indicating that gas6 inhibited chemotaxis and adhesion between monocytes and endothelial cells were inconsistent with other findings. Tjwa et al^33^ found that gas6 promoted leucocyte sequestration on the endothelium. Gas6^‐/‐^ mice were injected with TNF‐α to investigate sepsis and transplantation‐induced organ destruction, considering the organismic influence brought on by gas6 knockout, it is hard to attribute this effect to endothelial cells alone. Moreover, leucocytes sequestrated on the endothelium were not further discerned or classified—while it is clearly evident in our research that the recruitment of monocytes (a kind of the leucocyte) was inhibited by gas6 in HUVECs. Additionally, gas6 was reported to promote monocyte recruitment in venous thrombosis,^34^ gas6 is also expressed in platelets and interacts with endothelial cells, monocytes, and neutrophils. Cytokines secreted by platelets are stored in α‐granules, facilitate leucocyte recruitment and participate in thrombosis.^35^ Therefore, the involvement of gas6 from platelets in thrombosis cannot be ruled out. Considering the role of gas6 in immune and vascular system development^36^ and that macrophages in adult mice lacking TAM receptors were constitutively activated,^37^ the possibility that gas6 directly affects monocyte function should not be dismissed. Additionally, gas6 was also reported to augment ICAM‐1 and E‐selectin expression in human aortic endothelial cells induced by plasma membrane‐derived microparticles (PMPs),^38^ PMPs were shown to have pro‐inflammatory effects on the endothelium and PMPs can bind gas6, the alleged pro‐inflammatory effect of gas6 may be attributable to more stabilized and concentrative PMPs caused by gas6 binding.

To date, three receptors (Tyro3, Axl and Mer) of gas6 have been found. Axl and Mer have both been expressed in HUVECs,^38^ whether Tyro3 is also expressed in HUVECs remains to be determined. Tyro3 expression has not been detected in HUVECs via flow cytometry,^38^ but was observed at the mRNA level in Tjwa's study.^33^ A Western blotting assay was adopted in our studies. The monocytes group was used as a positive control,^39^, ^40^ and results indicated that no Tyro3 expression was detected in HUVECs, precluding further analysis of the Tyro3 receptor. As the functions of TAM receptors are context‐specific and independent,^41^ selective inhibitors of two receptors were introduced to understand which one was involved in the gas6 inhibitory effect. Results of ICAM‐1 and E‐selectin protein expression demonstrated that both receptors participate to mediate the effect. Imperfectly, the activation of receptors (ie the phosphorylated forms of the receptors), on cell membrane was not observed.

Earlier studies have shown that TAM inhibition of inflammation is transduced through the type I interferon receptor (IFNAR) and its associated transcription factor STAT1^15^; overlapping mechanisms for the inhibitory effect of gas6 likely exist. TAM receptor tyrosine kinases can directly recruit PI3 kinase and activate downstream Akt,^42^ thus PI3k/Akt pathway may be involved in the function of gas6. Congruent with previous findings,^43^ our results indicate that the NF‐κB pathway—which directly regulated ICAM‐1, E‐selectin, MCP‐1 and IL‐8 expression^44^, ^45^, ^46^—was restrained by Akt activation. To further verify this mechanism, recombinant human gas6 protein was introduced into pre‐treated HUVECs, and similar changes in the HUVEC’s Akt and p65 levels were noticed. These results being superficial and preliminary, detailed interactions between AKT and proteins that mediate NF‐κB signalling were not further explored in this study. Up‐to‐date research has since revealed that Akt could down‐regulate signalling—by affecting events that occur between the IKKβ (inhibitor of nuclear factor kappa‐B kinase β) and NF‐κB activation in the MyD88‐dependent pathway, and IRF3 (interferon regulatory factor 3) activity in the TRIF‐dependent pathway^43^—thus providing interesting insights on which to base future research. Phosphorylated Akt levels were also shown to be under the influence of NF‐κB activation,^47^ a finding further validated by our study. Increased levels of phosphorylated Akt was observed in *P. gingivalis*‐LPS stimulated HUVECs; however, this effect was dampened after the NF‐κB pathway was blocked, suggesting a shared regulation mechanism between the Akt and NF‐κB pathways.

In tissues from periodontitis‐compromised patients, gas6 expression levels were decreased, which is incongruent with a previous study.^48^ Gas6 mRNA expression was detected at similar levels in that study; however, further investigation on gas6 protein level was absent. Considering the samples’ heterogeneity, gas6 protein expression was analysed in matched, non‐inflamed and inflammatory tissues (collected from the same patient at the same time). This further validated our finding that gas6 expression is decreased under inflammatory conditions. More robust evidence could be acquired by recruiting more patients and exploring protein levels of gas6 in blood.

Evidence that TLR ligands reduce gas6 expression via NF‐κB activation suggests that a bi‐directional feedback system exists between gas6 and inflammation.^49^ In the present study, *P. gingivalis*‐LPS also reduced gas6 expression via NF‐κB activation. To further understand how gas6 expression was affected by NF‐κB, we focused on two antisense RNAs (GAS6‐AS1 and GAS6‐AS2) that were reported to exert effects in gas6 expression.^50^, ^51^ Antisense RNA is non‐coding RNA that is complementary to its related mRNA and effectively regulates gene expression at the replication, transcription and translation levels.^52^ Gas6 expression was regulated by GAS6‐AS1 via antisense overlapping, forming an RNA duplex to protect gas6 mRNA from ribonuclease degradation.^50^ To uncover which antisense RNA was involved in the NF‐κB mediated down‐regulation of gas6 expression, we analysed the mRNA level changes of gas6, GAS6‐AS1 and GAS6‐AS2 in HUVECs after *P. gingivalis*‐LPS infection, level changes for the antisense RNAs were similar to the gas6 mRNA level changes. The effect of NF‐κB activation on antisense RNA expression was observed, the reduced GAS6‐AS2 expression induced by LPS was reversed by NF‐κB inhibition, while GAS6‐AS1 expression remained unaffected. Therefore, GAS6‐AS2 might be the molecule connecting NF‐κB and gas6. To verify this hypothesis, three different GAS6‐AS2 shRNAs were introduced to knock‐down gas6 expression, which was significantly inhibited as expected. Furthermore, GAS6‐AS2 was unaffected when gas6 expression was altered using siRNA or plasmids, indicating that GAS6‐AS2 was an upstream regulator of gas6. These results together indicated that NF‐κB activation diminished gas6 via down‐regulating GAS6‐AS2 rather than GAS6‐AS1 expression. This information warrants further studies on the detailed interactions between NF‐κB and GAS6‐AS2. To our knowledge, this is the first evidence regarding the detailed mechanisms about how gas6 expression is regulated by NF‐κB activation.

**Figure 6:**
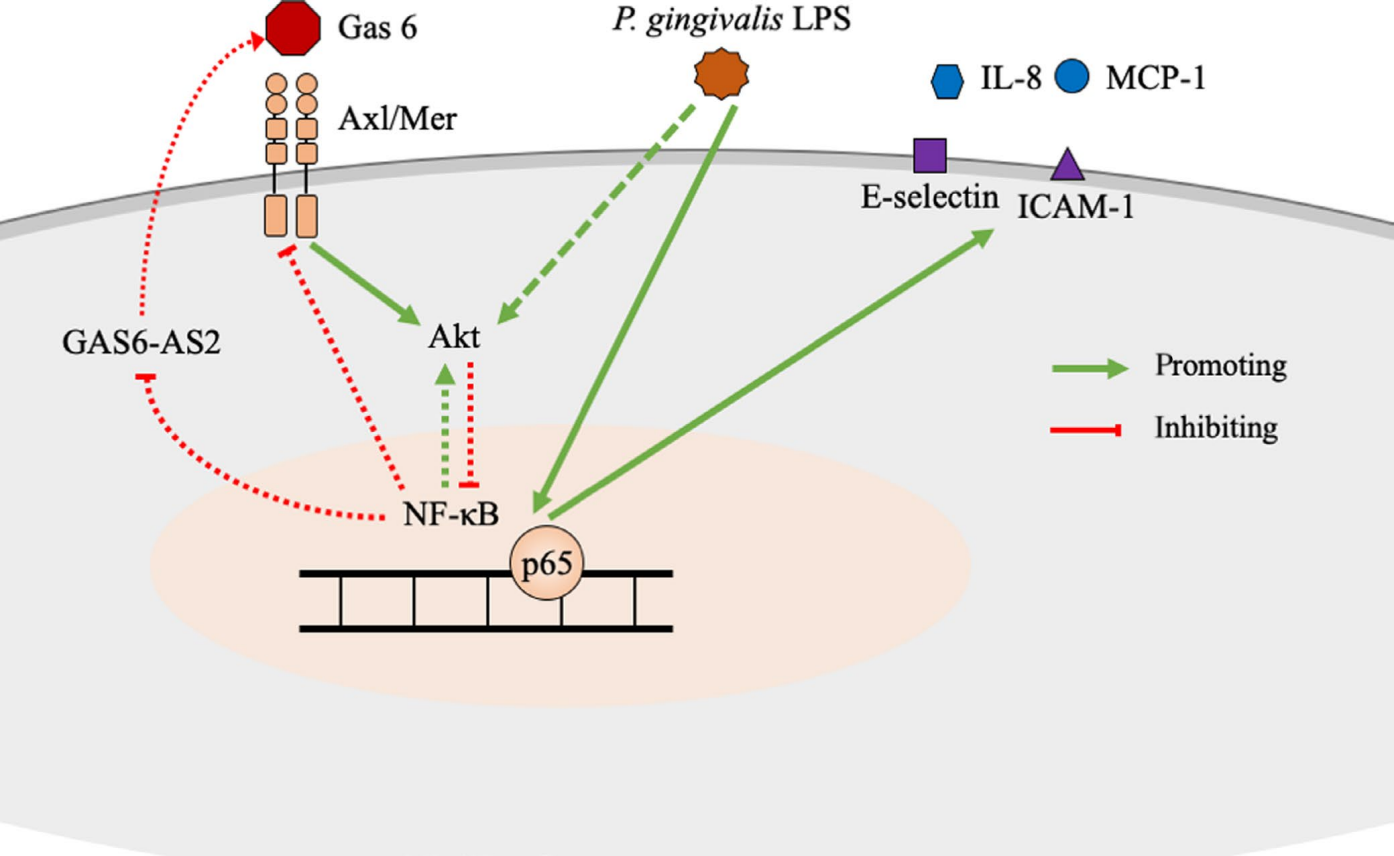
Schematic representation for mechanisms of bi‐directional regulation between gas6 and *P. gingivalis*‐LPS in HUVECs. Expression of MCP‐1, IL‐8, ICAM‐1 and E‐selectin induced by *P. gingivalis*‐LPS was inhibited by gas6 via Akt/NF‐κB pathway; Gas6 expression in HUVECs was inhibited by *P. gingivalis*‐LPS through NF‐κB/GAS6‐AS2 pathway

In summary, we observed that gas6 expression in HUVECs stimulated by *P. gingivalis*‐LPS inhibited the chemotaxis and adhesion of monocytes via the Akt/NF‐κB pathway. Moreover, gas6 expression was, in turn, inhibited by *P. gingivalis*‐LPS via NF‐κB activation, while LncRNA GAS6‐AS2 mediated the inhibitory effect of NF‐κB activation on gas6 expression (Figure 6). Further studies regarding effect of gas6 on periodontitis and atherosclerosis in vivo may endow us with novel insights into the connection between these two diseases.

## CONFLICT OF INTEREST

The authors confirm that there are no conflicts of interest.

## AUTHOR CONTRIBUTION

**Xuekui Wang:** Data curation (lead); Investigation (lead); Methodology (supporting); Project administration (supporting); Resources (supporting); Software (supporting); Validation (supporting); Visualization (supporting); Writing‐original draft (lead); Writing‐review & editing (supporting). **Yingjun Liu:** Data curation (lead); Investigation (supporting); Project administration (supporting); Validation (supporting); Writing‐original draft (supporting); Writing‐review & editing (lead). **Shengnan Zhang:** Conceptualization (supporting); Data curation (supporting); Formal analysis (supporting); Investigation (supporting); Methodology (supporting); Resources (supporting); Software (supporting); Visualization (supporting); Writing‐original draft (supporting). **Xiangying Ouyang:** Conceptualization (supporting); Funding acquisition (supporting); Resources (supporting); Supervision (supporting); Validation (supporting); Writing‐review & editing (supporting). **Yuguang Wang:** Investigation (supporting); Methodology (supporting); Project administration (supporting); Resources (supporting); Validation (supporting); Visualization (supporting). **Yong Jiang:** Resources (supporting); Software (lead); Supervision (supporting); Validation (supporting); Visualization (supporting). **Na An:** Conceptualization (lead); Data curation (supporting); Funding acquisition (lead); Investigation (lead); Methodology (lead); Project administration (lead); Resources (lead); Software (lead); Supervision (lead); Validation (lead); Visualization (lead); Writing‐original draft (supporting). AN came up with the research idea, study design and concept and supervised all aspects of the study. WXK and LYJ performed experiments. WXK and ZSN analysed and interpreted data and wrote the manuscript. WYG, OYXY and JY contributed to statistical analysis, participated in its design and coordination and helped to draft the manuscript. All authors read and approved the final manuscript.

## Supporting information

Supplementary Material

## Data Availability

The data that supports the findings of this study are available in the supplementary material of this article.

## References

[1] Pihlstrom BL , Michalowicz BS , Johnson NW . Periodontal diseases. Lancet. 2005;366(9499):1809‐1820. 16298220 10.1016/S0140-6736(05)67728-8

[2] Tonetti MS , Van Dyke TE . Periodontitis and atherosclerotic cardiovascular disease: consensus report of the Joint EFP/AAP Workshop on periodontitis and systemic diseases. J Clin Periodontol. 2013;40(Suppl 14):S24‐S29. 23627332 10.1111/jcpe.12089

[3] Suh JS , Kim S , Bostrom KI , et al. Periodontitis‐induced systemic inflammation exacerbates atherosclerosis partly via endothelial‐mesenchymal transition in mice. Int J Oral Sci. 2019;11(3):21. 31257363 10.1038/s41368-019-0054-1 PMC6802639

[4] Carrizales‐Sepulveda EF , Ordaz‐Farias A , Vera‐Pineda R , Flores‐Ramirez R . Periodontal disease, systemic inflammation and the risk of cardiovascular disease. Heart Lung Circ. 2018;27(11):1327‐1334. 29903685 10.1016/j.hlc.2018.05.102

[5] Macedo Paizan ML , Vilela‐Martin JF . Is there an association between periodontitis and hypertension? Curr Cardiol Rev. 2014;10(4):355‐361. 24739001 10.2174/1573403X10666140416094901 PMC4101200

[6] An N , Ou‐yang XY , Han W . Detection of periodental pathogens in atherosclerotic lesions in patients. Beijing Da Xue Xue Bao Yi Xue Ban. 2010;42(1):33‐36. 20140039

[7] Macedo Paizan ML , Vilela‐ Martín JF . Is There an association between periodontitis and hypertension? Curr Cardiol Rev. 2014;10(4):355‐361. 24739001 10.2174/1573403X10666140416094901 PMC4101200

[8] Gimbrone MA Jr , Garcia‐Cardena G . Endothelial cell dysfunction and the pathobiology of atherosclerosis. Circ Res. 2016;118(4):620‐636. 26892962 10.1161/CIRCRESAHA.115.306301 PMC4762052

[9] Kallio KA , Hatonen KA , Lehto M , et al. Endotoxemia, nutrition, and cardiometabolic disorders. Acta Diabetol. 2015;52(2):395‐404. 25326898 10.1007/s00592-014-0662-3

[10] Bhagat K , Moss R , Collier J , Vallance P . Endothelial "stunning" following a brief exposure to endotoxin: a mechanism to link infection and infarction? Cardiovasc Res. 1996;32(5):822‐829. 8944812

[11] Tonetti MS , D'Aiuto F , Nibali L , et al. Treatment of periodontitis and endothelial function. N Engl J Med. 2007;356(9):911–920. 17329698 10.1056/NEJMoa063186

[12] An N , Andrukhov O , Tang Y , et al. Effect of nicotine and porphyromonas gingivalis lipopolysaccharide on endothelial cells in vitro. PLoS One. 2014;9(5):e96942. 24820118 10.1371/journal.pone.0096942 PMC4018363

[13] Huang M , Rigby AC , Morelli X , et al. Structural basis of membrane binding by Gla domains of vitamin K‐dependent proteins. Nat Struct Biol. 2003;10(9):751‐756. 12923575 10.1038/nsb971

[14] Rothlin CV , Carrera‐Silva EA , Bosurgi L , Ghosh S . TAM receptor signaling in immune homeostasis. Annu Rev Immunol. 2015;33:355‐391. 25594431 10.1146/annurev-immunol-032414-112103 PMC4491918

[15] Rothlin CV , Ghosh S , Zuniga EI , et al. TAM receptors are pleiotropic inhibitors of the innate immune response. Cell. 2007;131(6):1124‐1136. 18083102 10.1016/j.cell.2007.10.034

[16] Nassar M , Tabib Y , Capucha T , et al. GAS6 is a key homeostatic immunological regulator of host‐commensal interactions in the oral mucosa. P Natl Acad Sci USA. 2017;114(3):E337‐E346. 10.1073/pnas.1614926114 PMC5255577 28049839

[17] Hurtado B , Munoz X , Recarte‐Pelz P , et al. Expression of the vitamin K‐dependent proteins GAS6 and protein S and the TAM receptor tyrosine kinases in human atherosclerotic carotid plaques. Thromb Haem. 2011;105(5):873‐882. 10.1160/TH10-10-0630 21384080

[18] Thorp E , Cui D , Schrijvers DM , et al. Mertk receptor mutation reduces efferocytosis efficiency and promotes apoptotic cell accumulation and plaque necrosis in atherosclerotic lesions of apoe‐/‐ mice. Arterioscler Thromb Vasc Biol. 2008;28(8):1421‐1428. 18451332 10.1161/ATVBAHA.108.167197 PMC2575060

[19] Holden RM , Hetu MF , Li TY , et al. Circulating Gas6 is associated with reduced human carotid atherosclerotic plaque burden in high risk cardiac patients. Clin Biochem. 2019;64:6‐11. 30508521 10.1016/j.clinbiochem.2018.11.018

[20] Ni J , Lin M , Jin Y , et al. Gas6 attenuates sepsis‐induced tight junction injury and vascular endothelial Hyperpermeability via the Axl/NF‐kappaB signaling pathway. Front Pharmacol. 2019;10:662. 31263416 10.3389/fphar.2019.00662 PMC6585310

[21] Liu YJ , Ouyang XY , Wang YG , et al. Role of vitamin K‐dependent protein Gas6 in the expression of endothelial cell adhesion molecule‐1 and chemokines induced by *Porphyromonas gingivalis* lipopolysaccharide. Beijing Da Xue Xue Bao Yi Xue Ban. 2018;50(1):20‐25. 29483717

[22] Kocgozlu L , Elkaim R , Tenenbaum H , Werner S . Variable cell responses to *P gingivalis* lipopolysaccharide. J Dent Res. 2009;88(8):741‐745. 19734462 10.1177/0022034509341166

[23] Wang YX , An N , Ouyang XY . Molecular mechanism involved in adhesion of monocytes to endothelial cells induced by nicotine and Porphyromonas gingivalis‐LPS. Beijing Da Xue Xue Bao Yi Xue Ban. 2015;47(5):809‐813. 26474621

[24] Ribeiro MC , Peruchetti DB , Silva LS , et al. LPS induces mTORC1 and mTORC2 activation during monocyte adhesion. Front Mol Biosci. 2018;5:67. 30073169 10.3389/fmolb.2018.00067 PMC6058081

[25] Chanput W , Mes JJ , Wichers HJ . THP‐1 cell line: an in vitro cell model for immune modulation approach. Int Immunopharmacol. 2014;23(1):37‐45. 25130606 10.1016/j.intimp.2014.08.002

[26] Sun X , Guan H , Peng S , et al. Growth arrest‐specific protein 6 (Gas6) attenuates inflammatory injury and apoptosis in iodine‐induced NOD.H‐2(h4) mice. Int Immunopharmacol. 2019;73:333‐342. 31129420 10.1016/j.intimp.2019.04.038

[27] Peng CK , Wu CP , Lin JY , et al. Gas6/Axl signaling attenuates alveolar inflammation in ischemia‐reperfusion‐induced acute lung injury by up‐regulating SOCS3‐mediated pathway. PLoS One. 2019;14(7):e0219788. 31318922 10.1371/journal.pone.0219788 PMC6638944

[28] Hirschi KM , Chapman S , Hall P , et al. Gas6 protein induces invasion and reduces inflammatory cytokines in oral squamous cell carcinoma. J Oral Pathol Med. 2018;47(8):748‐754. 29856094 10.1111/jop.12738

[29] Wu G , McBride DW , Zhang JH . Axl activation attenuates neuroinflammation by inhibiting the TLR/TRAF/NF‐kappaB pathway after MCAO in rats. Neurobiol Dis. 2018;110:59‐67. 29196212 10.1016/j.nbd.2017.11.009 PMC5748011

[30] Avanzi GC , Gallicchio M , Bottarel F , et al. GAS6 inhibits granulocyte adhesion to endothelial cells. Blood. 1998;91(7):2334‐2340. 9516131

[31] Lee CH , Shieh YS , Hsiao FC , et al. High glucose induces human endothelial dysfunction through an Axl‐dependent mechanism. Cardiovasc Diabetol. 2014;13:53. 24572151 10.1186/1475-2840-13-53 PMC3941696

[32] Li M , Ye J , Zhao G , et al. Gas6 attenuates lipopolysaccharideinduced TNFalpha expression and apoptosis in H9C2 cells through NFkappaB and MAPK inhibition via the Axl/PI3K/Akt pathway. Int J Mol Med. 2019;44(3):982‐994. 31524235 10.3892/ijmm.2019.4275 PMC6657963

[33] Tjwa M , Bellido‐Martin L , Lin Y , et al. Gas6 promotes inflammation by enhancing interactions between endothelial cells, platelets, and leukocytes. Blood. 2008;111(8):4096‐4105. 18156494 10.1182/blood-2007-05-089565

[34] Laurance S , Bertin FR , Ebrahimian T , et al. Gas6 promotes inflammatory (CCR2(hi)CX3CR1(lo)) monocyte recruitment in venous thrombosis. Arterioscler Thromb Vasc Biol. 2017;37(7):1315‐1322. 28450294 10.1161/ATVBAHA.116.308925

[35] von Bruhl ML , Stark K , Steinhart A , et al. Monocytes, neutrophils, and platelets cooperate to initiate and propagate venous thrombosis in mice in vivo. J Exp Med. 2012;209(4):819‐835. 22451716 10.1084/jem.20112322 PMC3328366

[36] Burstyn‐Cohen T . TAM receptor signaling in development. Int J Dev Biol. 2017;61(3‐4‐5):215‐224. 28621419 10.1387/ijdb.160285tb

[37] Lemke G , Lu Q . Macrophage regulation by Tyro 3 family receptors. Curr Opin Immunol. 2003;15(1):31‐36. 12495730 10.1016/s0952-7915(02)00016-x

[38] Happonen KE , Tran S , Morgelin M , et al. The Gas6‐Axl protein interaction mediates endothelial uptake of platelet microparticles. J Biol Chem. 2016;291(20):10586‐10601. 27006397 10.1074/jbc.M115.699058 PMC4865908

[39] Malawista A , Wang X , Trentalange M , et al. Coordinated expression of tyro3, axl, and mer receptors in macrophage ontogeny. Macrophage (Houst). 2016;3:e1261. 27695708 10.14800/macrophage.1261 PMC5040214

[40] Barth ND , Marwick JA , Heeb MJ , et al. Augmentation of human monocyte responses to lipopolysaccharide by the protein S and Mer/Tyro3 receptor tyrosine kinase axis. J Immunol. 2018;201(9):2602‐2611. 30249810 10.4049/jimmunol.1800249 PMC6201838

[41] Zagorska A , Traves PG , Lew ED , et al. Diversification of TAM receptor tyrosine kinase function. Nat Immunol. 2014;15(10):920‐928. 25194421 10.1038/ni.2986 PMC4169336

[42] Weinger JG , Gohari P , Yan Y , et al. In brain, Axl recruits Grb2 and the p85 regulatory subunit of PI3 kinase; in vitro mutagenesis defines the requisite binding sites for downstream Akt activation. J Neurochem. 2008;106(1):134‐146. 18346204 10.1111/j.1471-4159.2008.05343.x PMC2905062

[43] Zenke K , Muroi M , Tanamoto KI . AKT1 distinctively suppresses MyD88‐depenedent and TRIF‐dependent Toll‐like receptor signaling in a kinase activity‐independent manner. Cell Signal. 2018;43:32‐39. 29242168 10.1016/j.cellsig.2017.12.002

[44] Kunsch C , Rosen CA . NF‐kappa B subunit‐specific regulation of the interleukin‐8 promoter. Mol Cell Biol. 1993;13(10):6137‐6146. 8413215 10.1128/mcb.13.10.6137-6146.1993 PMC364673

[45] Ueda A , Okuda K , Ohno S , et al. NF‐kappa B and Sp1 regulate transcription of the human monocyte chemoattractant protein‐1 gene. J Immunol. 1994;153(5):2052‐2063. 8051410

[46] van de Stolpe A , Caldenhoven E , Stade BG , et al. 12‐O‐tetradecanoylphorbol‐13‐acetate‐ and tumor necrosis factor alpha‐mediated induction of intercellular adhesion molecule‐1 is inhibited by dexamethasone. Functional analysis of the human intercellular adhesion molecular‐1 promoter. J Biol Chem. 1994;269(8):6185‐6192. 7907090

[47] Meng F , Liu L , Chin PC , D'Mello SR . Akt is a downstream target of NF‐kappa B. J Biol Chem. 2002;277(33):29674‐29680. 12052823 10.1074/jbc.M112464200

[48] Jiang L , Chen XQ , Gao MJ , et al. The Pros1/Tyro3 axis protects against periodontitis by modulating STAT/SOCS signalling. J Cell Mol Med. 2019;23(4):2769‐2781. 30729671 10.1111/jcmm.14183 PMC6433735

[49] Deng T , Zhang Y , Chen Q , et al. Toll‐like receptor‐mediated inhibition of Gas6 and ProS expression facilitates inflammatory cytokine production in mouse macrophages. Immunology. 2012;135(1):40‐50. 22043818 10.1111/j.1365-2567.2011.03511.x PMC3246651

[50] Zhang P , Dong Q , Zhu H , et al. Long non‐coding antisense RNA GAS6‐AS1 supports gastric cancer progression via increasing GAS6 expression. Gene. 2019;696:1‐9. 30735718 10.1016/j.gene.2018.12.079

[51] Wen L , Zheng Y , Wen X , et al. Increased expression of long noncoding RNA GAS6‐AS2 promotes proliferation and inhibits apoptosis of melanoma cells via upregulating GAS6 expression. IUBMB Life. 2019;71(10):1503‐1514. 31162889 10.1002/iub.2071

[52] Xu JZ , Zhang JL , Zhang WG . Antisense RNA: the new favorite in genetic research. J Zhejiang Univ Sci B. 2018;19(10):739‐749. 30269442 10.1631/jzus.B1700594 PMC6194357

