# Supplementary figures and images for: Crosstalk between Akt and NF‐κB pathway mediates inhibitory effect of gas6 on monocytes‐endothelial cells interactions stimulated by P. gingivalis‐LPS
Source: J Cell Mol Med. 2020 May 28;24(14):7979–90. doi: 10.1111/jcmm.15430 (PMC7348146; doi:10.1111/jcmm.15430)

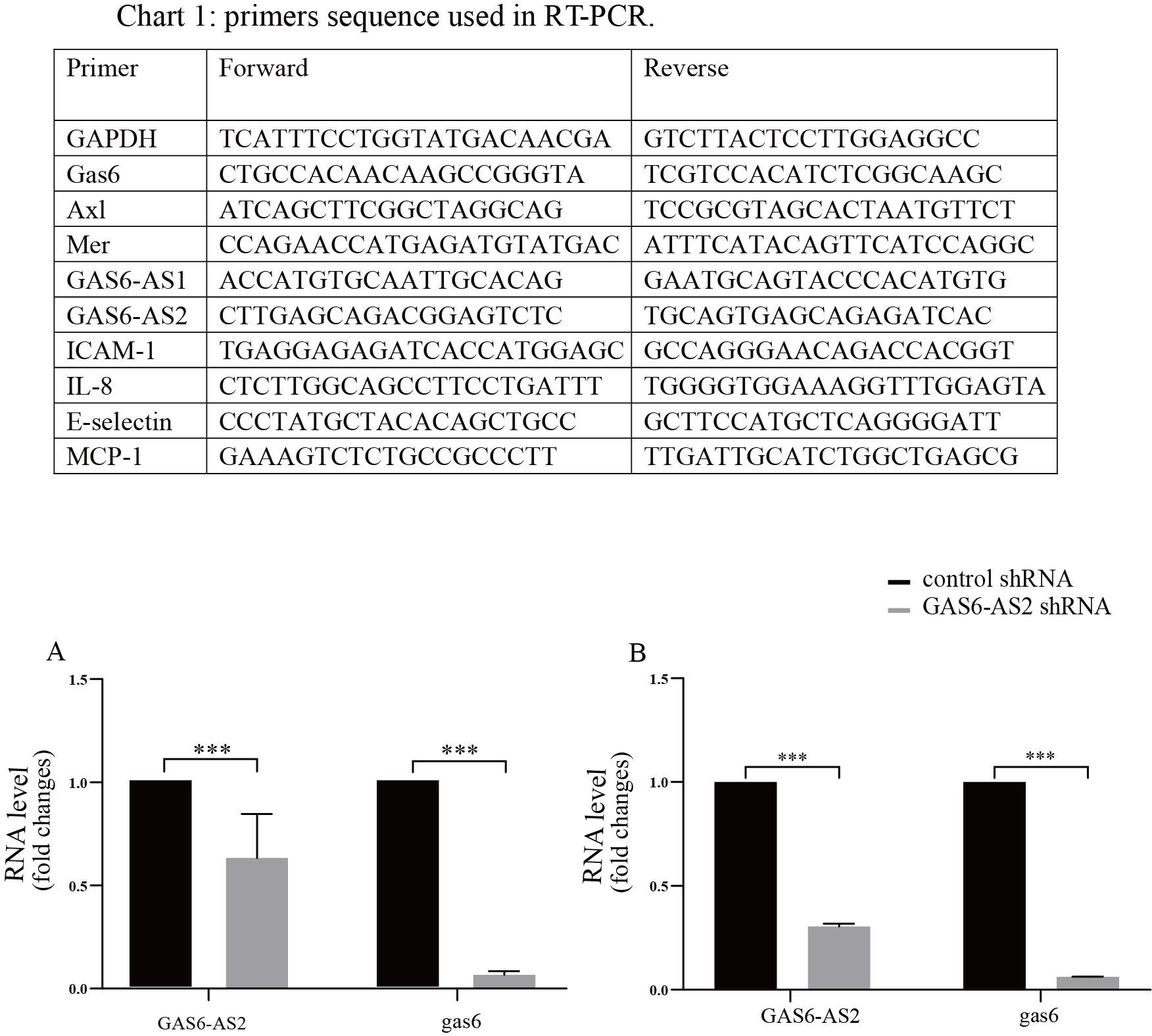

Supplement: Supplementary file 1 — Supplementary Material [file JCMM-24-7979-s001.png]
